# Benign Biliary Strictures: A Comprehensive Review

**DOI:** 10.5152/tjg.2024.24044

**Published:** 2024-07-01

**Authors:** Munish Ashat, Joseph Berei, Rami El-Abiad, Mouen A. Khashab

**Affiliations:** 1 Division of Gastroenterology and Hepatology, University of Iowa Hospitals and Clinics, Iowa City, Iowa, USA; 2 Department of Internal Medicine, University of Iowa Hospitals and Clinics, Iowa City, Iowa, USA; 3 Division of Gastroenterology and Hepatology, Johns Hopkins Hospital, Baltimore, Maryland, USA

**Keywords:** Biliary stricture, cholangioscopy, ERCP, fluorescence in situ hybridization, next generation sequencing

## Abstract

Benign biliary strictures (BBS) ensue from inflammatory conditions (e.g., chronic pancreatitis) or post surgery (e.g., cholecystectomy and liver transplant). High-quality cross-sectional imaging studies such as computed tomography or magnetic resonance cholangiopancreatography are essential in the diagnosis and planning of therapeutic interventions and in ruling out malignancy. Endoscopic retrograde cholangiopancreatography with dilation and stenting is the mainstay treatment for BBS, while surgery is reserved for failed endoscopy or refractory cases.

**Main Points**

- Biliary stricture evaluation needs a multidisciplinary approach.
- Understanding the etiology and ruling out malignancy is essential before deciding on the optimal treatment approach.
- Benign strictures can be managed using plastic or covered self-expanding metal biliary stents.
- Indeterminate cases need endoscopic retrograde cholangiopancreatography biliary cytology and forceps biopsy, although these are limited by having low sensitivity.
- Additional modalities, including cholangioscopy, endoscopic ultrasound, fluorescence in situ hybridization, and next-generation sequencing, can provide additional information and increase diagnostic yield.

## Introduction

Benign biliary strictures (BBS) can result from surgical injuries, chronic inflammatory conditions, or infectious causes such as recurrent cholangitis. These diverse etiologies trigger local inflammatory responses and ischemic insults that effectuate secondary fibrosis and scarring, manifesting as cholestasis. Bile duct injury during cholecystectomy is the most common cause of post-operative BBS, whereas chronic pancreatitis accounts for the majority of inflammatory BBS.^1^ Clinical presentation varies based on the etiology, chronicity, location, and degree of obstruction. It can range from cholestasis with mildly elevated liver chemistries, jaundice, pruritus, and bilirubinuria to recurrent cholangitis and, ultimately, secondary biliary cirrhosis. Biliary strictures, irrespective of cause, are diagnosed based on signs and symptoms of biliary obstruction, as corroborated by focal upstream biliary ductal dilation on abdominal imaging. Ultrasound and computed tomography (CT) can help identify strictures through visualization of a transition point of a dilated common bile duct (CBD). Yet magnetic resonance cholangiopancreatography (MRCP) is superior in accurately delineating the anatomy of and localizing the stricture. In contrast, endoscopic retrograde cholangiopancreatography (ERCP) is integral for cytological/histopathological diagnosis and therapeutic interventions. In indeterminate cases, serum liver function tests and tumor markers such as carbohydrate 19-9 (CA 19-9), endoscopic ultrasound (EUS), and cholangioscopy may aid in identifying the underlying diagnosis and in excluding malignancy, although longitudinal follow-up may still be required.

Interventions are aimed at prompt and durable biliary drainage to preserve liver function and to prevent irreversible severe complications such as recurrent cholangitis or secondary biliary cirrhosis. Bolstered by a variety of accessories like stents, catheters, and guidewires, ERCP has emerged as the preferred therapeutic modality for BBS owing to its relative safety, efficacy, and minimally invasive nature. Radiological approaches, on the other hand, can complement endoscopy in providing biliary access or decompression, while surgery acts as a salvage therapy for refractory strictures.

This review will discuss the etiology and classification of BBS and focus on BBS management and outcomes in specific scenarios.

### Classification

Their anatomical classification guides the management strategy for BBS according to 2 commonly utilized systems. The Bismuth classification (Table 1) is based on the location of the stricture and whether or not the bile duct bifurcation or individual right sectoral duct is involved.^2^ This classification system, devised in the era of open surgery, aims to identify the interface between the most distal level of healthy biliary mucosa and that of the proximal injury site, which is crucial for successful anastomosis. In addition, the Bismuth classification serves as a tool for determining prognosis post repair. The Strasberg classification comments on bile leakage and elaborates on the Bismuth classification by describing the stricture anatomy, size, and location.^1^ On top of incorporating Bismuth’s classification (type E subclasses), the Strasberg classification (Table 2) also includes additional types of laparoscopic injuries to the extrahepatic duct.^3^ As such, some favor this comprehensive classification as it allows for differentiation between minor and severe injuries sustained in laparoscopic cholecystectomy.^3^ It is generally accepted that strictures involving the CBD or distal common hepatic duct are less complicated to repair and have lower recurrence rates compared to more proximal counterparts.^3^

### Diagnosis

The diagnosis of BBS is entertained in patients with pertinent history, cholestatic labs, and suggestive imaging (biliary ductal dilation with a transition point). Imaging studies, including CT and/or MRI/MRCP with and without contrast, can rule out other etiologies (such as gallstones) and help provide a roadmap for endoscopic management. A multidisciplinary approach can guide the therapeutic plan in indeterminate cases. Magnetic resonance imaging/MRCP is superior in delineating the location and length of biliary strictures and may help identify malignant biliary strictures (MBS). When histological diagnosis is required, ERCP with brush cytology and/or forceps biopsy is pursued.

A meta-analysis demonstrated 45% sensitivity and 99% specificity for ERCP with brush cytology in diagnosing MBS. Similarly, a low sensitivity of 48.1% and specificity of 99.2% were noted with forceps biopsies. Invariably, the sensitivity was 59.4% and specificity 100% when a combination of cytology and forceps biopsy were employed.^4^ Consequently, additional diagnostic modalities like EUS-guided fine needle biopsy, cholangioscopy-guided biopsies, confocal laser electron microscopy, fluorescence in situ hybridization (FISH), and/or next-generation sequencing (NGS) may be needed to differentiate MBS from BBS. If the diagnosis remains doubtful, patients should be followed for at least 6 months to confirm a benign course. In a prospective single-center study, 36.5% of 104 patients with indeterminate strictures were diagnosed with cancer in the first 6 months of follow-up. Notably, owing to a higher risk of cholangiocarcinoma, enrolling primary sclerosing cholangitis (PSC) patients in a lifelong surveillance program is prudent.^5^

## Management

### Endoscopic

Among the available modalities, ERCP remains the mainstay procedure in managing BBS. It offers biliary access to diagnostics and therapeutics with an acceptable safety profile. The therapy goals are adequate drainage and durable stricture dilation, which are met with several challenges, such as stent migration and cholangitis. Recurrence of the stricture, either due to inadequate treatment or secondary to reactive hyperplasia to an indwelling stent, can also delay or falter success.

Endoscopic dilation of biliary strictures is performed during ERCP after passing an ERCP wire beyond the stricture, followed by an appropriately sized balloon catheter. The diameter of the duct distal to the stricture dictates the choice of a suitable balloon. The balloon is then distended under fluoroscopic guidance until the effacement of its waist and the radial force is maintained for 30-60 seconds. A less aggressive approach is recommended when dilating within 30 days post biliary anastomosis as this carries a higher risk of dehiscence/leak.^6,7^ Given that balloon dilation without stent placement is associated with rates of restenosis as high as 47%, concurrent stent placement post balloon dilation is recommended.^8^

A notable exception to this general rule is in patients with PSC-related strictures. Either dilation alone or dilation followed by short-term stenting (e.g., 2 weeks) are preferred to avoid chronic bacterial contamination of the biliary tree.

Owing to their ease of use, especially regarding removability, plastic stents are the stents of choice when managing BBS. Plastic stents come in various shapes, lengths, diameters, construction materials, coatings, and anti-migration mechanisms. This offers versatility with a wide selection of choices suitable for most scenarios. However, should stents longer than 15 cm become necessary, nasobiliary drains can be fashioned to the required length albeit at increased migration risk.

To achieve adequate biliary stricture dilation and to overcome anticipated stent blockage, multiple plastic stents are sequentially inserted alongside each other every 3-4 months throughout the 12 to 18-month follow-up.^9^ Single-session placement of several juxtaposed plastic stents reduces the need for multiple ERCPs while improving long-term outcomes of BBS compared to using single or 2 stents.^9,10^ Despite anti-migration flaps, plastic stents are still prone to migration in 5%-10% of cases, and given their relatively smaller caliber, stent occlusion is observed 30% of the time, mainly when a single stent is utilized.^11,12^ Placement of multiple plastic stents decreases the risk of biliary obstruction due to stent occlusion as it allows bile flow through the lumens of multiple stents and between (wicking) stents. Placement of as many plastic stents as technically possible diminishes the risk of stricture recurrence after stent removal.

To surmount the challenges of frequent ERCPs for exchanging occluded plastic stents, self-expanding metal stents (SEMS) have been employed to treat BBS. Self-expanding metal stents provide prompt intervention for longer indwelling time, thus reducing accrued endoscopy-related costs and improving patient compliance. With a streamlined delivery system and a larger luminal diameter, SEMS can offer immediate and ample stricture dilation with longer stent patency and fewer ERCPs than plastic stents.^13^ There are multiple types of SEMS: uncovered, partially covered, and fully covered. An ideal stent is a temporary stent that is migration resistant, occlusion proof, and easy and safe to retrieve after it has served its purpose, irrespective of indwell time. Ultimately, stricture resolution is contingent on a stable stent position—enough for tissue remodeling to mature. The uncovered portion of a stent— partial or complete—induces reactive tissue hyperplasia, which, in addition to making retrieval risky (and sometimes impossible), can contribute to early stent occlusion. Ostensibly, this may be a disadvantage in the case of BBS. Fully covered SEMS, on the other hand, can relatively be removed safely after a prolonged indwell time. However, despite a rate of stricture resolution approaching 60%-100 % at the time of stent removal, the success is marred by a migration rate that correlates with the underlying indication. Stent migration may be partial or complete and is affected by the location of the stricture and its cause. Still, stent migration negatively impacts the resolution success of any BSS. In a nonrandomized multinational study including 177 patients, the stricture resolution rate, regardless of etiology, was 90.1% among patients undergoing scheduled fully covered (FC)-SEMS removal, 36% requiring early removal, and 50% experiencing complete distal migration. Fully covered SEMS migration was reported in 55 patients, with 36.4% experiencing partial distal dislocation, 29.1% complete, and 34.5% proximal migration.^14^ Chronic pancreatitis patients had the lowest migration rates of less than 5% through 6 months, and stent removal was the most successful (80.5%) compared to post-liver transplant (63.4%) and post-cholecystectomy strictures (61.1%) after a median indwell time of 11.3 months, 5.0 months, and 11.5 months respectively.

### Newer Modalities

In recent years, we have witnessed the revival of older methods and the utilization of available modalities/accessories in a manner that is befitting for BBS. Magnetic compression anastomosis (MCA), radiofrequency ablation (RFA), and biodegradable stents offer salvage solutions for unique, onerous cases. Although still considered experimental, MCA is a viable option for BBS that is refractory and difficult to treat with conventional methods, especially in cases of complete ductal obstruction or when the guidewire cannot be passed across a tight stricture. The concept relies on constructing a suture-less anastomosis driven by ischemic tissue compression.^15^ It takes about 53.3 days (range 9-181 days) for the bilio-biliary anastomosis to mature.^16^ Still, the time to remove the magnets depends on the distance between the magnets and the magnetic field strength of the magnets used. A combined percutaneous transhepatic biliary drainage (PTBD) (>16 Fr) and ERCP procedure are required to deliver the magnets. Although the scope techniques have been described,^17,18^ placing magnets via ERCP across the major papilla can prove challenging, and the placement of FC-SEMS may become necessary. Multiple experimental and clinical studies have looked at the feasibility and safety of MCA in BBS, although long-term outcomes remain lacking.^16,19-21^ In a study of 39 patients undergoing MCA for the management of post-traumatic or postoperative strictures and over a mean follow-up period of 41.9 months (range 7.1 to 73.4 months), recanalization was achieved in 35 patients. One patient developed cholangitis, and restenosis occurred in 1 patient, but no procedure-related mortality was reported.^22^

Radiofrequency ablation uses a Habib^TM^ EndoHPB (London, UK) probe to deliver 10 W energy over 90 seconds via a propriety RF generator (ESG 100; Olympus, Tokyo, Japan). In a pilot feasibility study, 9 patients with BBS (post-operative n = 4, liver transplant n = 3, chronic inflammation n = 2) who failed previous endoscopic and percutaneous dilations were enrolled. Intraductal RFA was delivered to each stricture segment, followed by balloon dilation with or without stenting. Four of 5 patients had no stricture recurrence at a median follow-up of 12.6 months.^23^ In a subset of patients where standard endoscopic therapy has failed, PTBD or surgical bypass may prove necessary. With PTBD, and when plastic stents are placed, frequent stent exchanges are required, making the procedure cumbersome and costly. In such scenarios, using biodegradable stents can be an attractive and reasonable alternative. In a retrospective study, 107 patients with BBS who underwent percutaneous biodegradable stent placement were followed for a median of 16 months. Of the 105 patients who experienced immediate stent dislodgement, adequate clinical response was observed in 90% of cases. Nineteen patients (18%) had stricture recurrence at a mean interval of 15.4 months.^24^ Similar results were reported in a small series of 13 patients in which biodegradable stents were endoscopically placed, and stricture resolution occurred in 83% of cases at 21-month follow-up.^25^

## Etiology and Endoscopic Management

### Chronic Pancreatitis 

**Figure 1.**
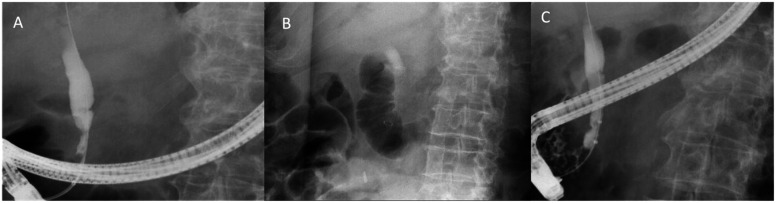
Distal bile duct stricture related to chronic pancreatitis stricture (A). Fully covered-self-expanding metal stents (FC-SEMS) placement (B). Improvement in biliary stricture post FC-SEMS removal (C).

In chronic pancreatitis (Figure 1), and owing to the native anatomical location, distal bile duct strictures develop secondary to the scarring and fibrosis from recurrent/chronic inflammation, especially that emanating from the pancreatic head. Benign biliary strictures has been reported in 3% to 23% of patients with chronic fibrosing pancreatitis chronic fibrosing pancreatitis (CFP).^26^ CFP patients with abnormal cholestatic liver chemistries should be screened for pancreatic malignancy with dedicated imaging (CT or MRI/MRCP) due to their 16-fold increased risk within the first 2 years of diagnosis compared to normal controls. Although this risk declines over time, it remains elevated compared to the average patient population. It is 8-fold at 5 years and 3-fold at a 9-year lag period from chronic pancreatitis diagnosis.^27^ Most asymptomatic elevation in alkaline phosphatase (ALP) and total bilirubin (≥2 upper limit of normal [ULN]) resolves within 1 month. However, with persistent elevation, biliary decompression becomes necessary because the incidence of secondary biliary cirrhosis can reach 7%.^28^ It is an acceptable practice to offer ERCP to patients with suspected BBS with persistently elevated liver chemistries (≥4 weeks).^26^ Multiple modalities are available to manage BBS in chronic pancreatitis, including placement of biliary stents [plastic (single vs. multiple side-by-side plastic stents), SEMS, or surgical bypass. Compared to surgical bypass, endoscopic stenting is associated with lower procedural morbidity (83 % vs. 21%) and a lower success rate at 2 years (66 % vs. 15%).^29^ Since surgical outcomes in patients post endoscopic intervention are similar to those who are treatment naïve, endoscopy is the first-line treatment while surgery is reserved for failed cases. The European Society for Gastrointestinal Endoscopy (ESGE) recommends against using single plastic stents or uncovered SEMS for managing BBS, given poor long-term results.^30^ When comparing multiple side-by-side plastic stents vs. covered SEMS (cSEMS), a meta-analysis including 4 studies with 213 patients showed similar outcomes for stricture resolution [1.07 (0.97 - 1.18)] and adverse events [1.16 (0.71 - 1.88)]. A higher cost was associated with using cSEMS despite fewer ERCPs required (95%CI −2.34 to −1.09).^31^ When choosing a plastic stent, an indwell stenting period of at least 12 months is recommended.^32^ Whereas when utilizing cSEMS, a fully covered SEMS is favored over a partially cSEMS, and an anticipated indwell time of 4-6 months on average is necessary to achieve a durable response.^33^ Stent migration is the most common complication, with cSEMS occurring in 9% of cases.^33^ Failure of endoscopic treatment is more common in the setting of pancreatic head calcifications. One study showed that only 7.7% of patients with pancreatic head calcifications and biliary obstruction had a clinical response to biliary stenting at 1-year follow-up.^32^ As such, surgical bypass becomes a reasonable alternative for this patient subset.

### Postoperative Benign Biliary Stricture

Post-cholecystectomy biliary strictures remain the most common cause of post-operative BBS. About a quarter of these BBS can be attributed to ductal injury in a background of aberrant anatomy where the right anterior or posterior ducts join the bile duct near the cystic duct.^34^ Injuries during cholecystectomy can result from direct ductal trauma following inadvertent transection from clipping or ligation or via ischemia secondary to dissection-related thermal injury. Early postoperative BBS can be related to bile leaks. The laparoscopic technique for cholecystectomy has been associated with an increase in bile duct injuries between 0.1 and 0.6% when compared to previously studied open cholecystectomy surgery.^34-36^ Surgical repair, namely hepaticojejunostomy, was the standard treatment for BBS; however, endoscopic dilation with stent placement has become the preferred alternative with similar clinical outcomes. Excellent or good long-term outcomes were observed in 80% of endoscopically treated post-cholecystectomy-related BBS vs. 77.3% in those surgically repaired.^37^ The location of the bile duct injury plays a vital role in selecting the type (plastic vs. SEMS) and number of plastic stents needed. As a general rule, FC-SEMS are used if the BBS is more than 2 cm from the hepatic confluence, whereas plastic stents are used if BBS is intrahepatic, or the hepatic hilum is involved. For multisegmental ischemic strictures, multiple plastic stents are needed.

### Post-Liver Transplant Stricture

**Figure 2.**
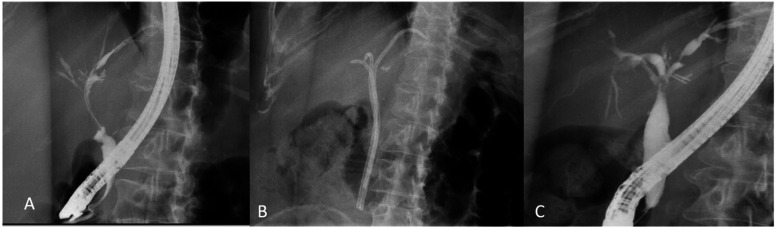
Post-liver transplant anastomotic stricture (A). Multiple plastic stents were placed to dilate the stricture (B) gradually. Post-stent removal improvement in anastomotic stricture (C).

Strictures and bile leaks are the most common biliary complications after liver transplantation, with a BBS reported incidence between 4% and 43% (Figure 2).^38^ Like chronic pancreatitis-related BBS, post-transplant strictures are initially treated with endoscopic therapy with surgical bypass reserved for failed or complicated cases. Post-transplant biliary strictures can be separated into anastomotic and non-anastomotic groups. Strictures occurring shortly after surgery (within 1 month) are generally related to perioperative events such as dissection or cautery-related injury—akin to cholecystectomy-related injury—and are more likely to be anastomotic in origin. On the other hand, biliary strictures occurring at or greater than 5 mm proximal to the anastomosis are defined as non-anastomotic biliary strictures (NABS) and are generally caused by ischemia. Often, the ischemia is secondary to hepatic artery stenosis or thrombosis, which is the sole blood supplier to the biliary tree.^39^ This stricture type typically occurs at the hilum but may progress to the intrahepatic ducts.^40^ Non-anastomotic biliary strictures generally occurs earlier post-operatively compared to ABS, with a 3 to 6 months average time until stricture diagnosis.^41,42^

Endoscopic treatment of anastomotic biliary strictures (ABS) with balloon dilation and stent placement is successful in 67% to 100% of cases.^43-46^ It is expected that ABS occurring 6 months or later post-transplantation may require more endoscopic interventions than strictures manifesting earlier.^47^ Furthermore, endoscopic therapy is not as effective for NABS as it is for ABS, with estimated success rates ranging between 40 and 82%.^48,49^ Non-anastomotic biliary strictures often requires repeat episodes of dilation and prolonged periods of stenting and is associated with higher rates of stricture recurrence.

### Primary Sclerosing Cholangitis

**Figure 3.**
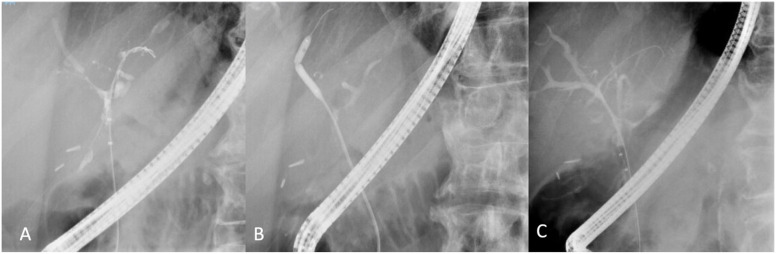
Primary sclerosing cholangitis stricture before balloon dilation (A). Multi-segmental balloon dilation was performed using a 4 mm balloon (B). Stricture improvement seen post balloon dilation (C).

Primary sclerosing cholangitis (Figure 3) is a chronic progressive inflammatory cholestatic liver disease with intra and/or extrahepatic biliary strictures that can be associated with inflammatory bowel disease. Magnetic resonance cholangiopancreatography has become the diagnostic tool of choice and has replaced ERCP in this role. Endoscopic retrograde cholangiopancreatography in PSC patients is associated with increased hospitalization rates reaching 10%, as well as bacterial cholangitis, bile duct perforation, and pancreatitis. Yet, ERCP still serves a well-defined role in specific PSC scenarios:

1. To identify a subset of patients with indeterminate and suboptimal intrahepatic images on MRCP for diagnosing PSC.
2. To evaluate and treat previously stable PSC patients with worsening pruritus, liver chemistries, or bacterial cholangitis
3. To rule out cholangiocarcinoma in patients with dominant strictures, defined as a CBD diameter of 1.5 mm or less or right and left hepatic ducts with a diameter of 1 mm or less.

Balloon dilation of benign strictures with or without stenting in PSC remains the first-line therapy.^50^ Multiple sessions are usually required over a short interval to achieve the desired clinical success, although data regarding the ideal balloon diameter still needs to be settled. The ESGE recommends a 10 Fr stent for CBD strictures and two 7 Fr stents if bilateral intrahepatic stents are required.^51^ In the most extensive study involving 96 patients undergoing 500 endoscopic balloon dilations, 8 mm balloons were utilized for extrahepatic ducts, and 6-8 mm balloons for intrahepatic ducts. Dilations were done at 1-4 weeks intervals, and 2-3 sessions were performed on average.^52^ Eighty-one percent of patients met the end goal of liver transplant-free survival at 5 years, and 52% met the same goal at 10 years.^52^ In the absence of adequate clinical response to balloon dilation alone, short-term stenting can be considered as similar efficacy has been observed between short-term stenting (1-2 weeks) and standard (8-12 weeks) stenting, albeit with higher occlusion rates in the latter group.^53^ It is important to note that antibiotic prophylaxis is universally recommended for PSC patients undergoing ERCP,^50^ although the duration remains arbitrary.

### Immunoglobulin G4-Related Cholangiopathy

Immunoglobulin G4-related cholangiopathy (IgG4-SC)—a subset of systemic IgG4-related diseases—is manifested by increased IgG-4 plasma cells, lymphocytosis, obliterative phlebitis, and fibrosis in the bile duct. Usually, IgG4-SC is associated with autoimmune pancreatitis, although it can be independently seen in up to 20% of cases.^54^ Steroids remain the mainstay therapy, and a prompt response may serve as a diagnostic confirmation of the suspected autoimmune disease. Temporary biliary stenting, however, can be performed while the patient is undergoing diagnostic workup or has contraindications to steroid therapy.

### Indeterminate Biliary Duct Strictures

Pancreaticobiliary tumors may present as biliary or pancreatic strictures without a visible mass on imaging. A similar plight can also be encountered in benign scenarios like autoimmune or chronic pancreatitis. These strictures, labeled indeterminate biliary strictures (IDBS), constitute a diagnostic dilemma as they remain unascertained despite a comprehensive workup, including laboratory testing, abdominal imaging, and ERCP with cytological brushing and/or biopsies. Brush cytology is among the first-line diagnostic tools in evaluating biliary strictures. When combined with forceps biopsy sampling, brush cytology has a sensitivity within the 44%-89% range and a specificity close to 100%.^55^ The low sensitivity may pose a diagnostic challenge, especially in the setting of malignancy, delaying prompt intervention and possibly a curative surgery. Analogously, it may subject patients to unwarranted surgeries, as observed in 15% of BBS cases.^56,57^

To overcome some of these challenges and to better detect bile duct malignancies manifesting as IDBS, several complementary techniques have been developed or added, including FISH, NGS, cholangioscopy-based tissue sampling, and EUS guided biopsies.

Fluorescence in situ hybridization is a molecular cytogenetic method that detects fluorescently labeled DNA or oligonucleotide probes hybridized to metaphase or interphase cells. The test, performed on samples collected from intraductal brushings, capitalizes on the fact that most solid tumors have abnormal numbers of chromosomes.^55^ The technology scrutinizes for aneuploidy (abnormal number of chromosomes) using fluorescently labeled probes targeting the centromeres of chromosomes 3,7, 17, and 9p21 band (P16) and provides quantitative data for cytopathological diagnosis. Positive results inferring malignancy include tetrasomy and polysomy of chromosomes 3, 7, or 17 and deletion of the 9p21 locus. Compared to routine brush cytology, FISH yielded a sensitivity of 35% to 52%, which improved to 84.2% when both modalities were combined (with a specificity of 54.1%).^55,58,59^

Furthermore, the same retrospective single-center study revealed that triple modality evaluation in 124 patients combining brush cytology, forceps biopsy, and FISH and employing extended diagnostic criteria, wherein suspicious and high-grade dysplasia were considered malignant, the sensitivity jumped to 92.6% (95% CI 75.5-99.1%) while specificity stalled at 51% (CI 43.4-58.6%).^60^ It is important to note that FISH is labor-intensive as nuclear overlap can lead to difficulties in probe copy enumerations. Fluorescence in situ hybridization is also prone to subjective interpretations, which could explain the differences in reported sensitivities.

Next-generation sequencing provides an unique opportunity to identify a small proportion of mutated cells using highly sensitive molecular assays in an otherwise small quantity of heterogeneous cells. Using samples collected during ERCP, NGS identifies deleted, mutated, and/or amplified genes commonly encountered in biliary malignancies. In a prospective single-center study enrolling 252 patients, NGS improved the sensitivity of brush cytology and biliary biopsies from 35% to 77% and 52% to 83%, respectively.^61^ This study had a false-negative rate of 25% (37 cases), attributed to inadequate sampling and/or low tumor cellularity. The absence of a standardized protocol that dictates what gene panel needs to be tested and how NGS is best integrated within the IBSD evaluation strategy has contributed to the heterogeneous results and the multiform practice.

Nonetheless, for an adjunctive test, NGS seems more sensitive than FISH, especially when integrated with cytology. In a study analyzing biliary brushing samples from 73 cases and pancreatic brushing samples from 8 cases, the 67% sensitivity from cytology alone improved to 76% when cytology was combined with FISH and to 85% when cytology was combined with NGS.^62^

Cholangioscopy facilitates direct observation of the biliary lumen and the associated strictures. Of the several available methods for cholangioscopic assessment, the most commonly utilized is SpyGlass Digital System™ manufactured by Boston Scientific. Features attributed to MBS include dilated and tortuous vessels, polypoid masses, oozing, and irregular mucosal surfaces,^63,64^ all of which may be prone to interobserver variability.^65^ Nevertheless, a retrospective study has found that the visual diagnosis of malignant strictures has a sensitivity of 88.9% and 97% and a specificity of 94.5% to 97.6%.^66^ Another retrospective study involving 614 patients who underwent ERCP to assess biliary strictures found that a combination of brush cytology and fluoroscopic biopsy (n = 259) was sensitive in 62.5% of cases (95% CI, 52.5-71.8%). In this study, 59 patients had triple modality testing using cytology, fluoroscopic biopsy, and cholangioscopic biopsies, which resulted in a sensitivity of 76.7% (95% CI 57.7-90.1%).^60^

Endoscopic ultrasound plays a vital role in evaluating IDBS, although its performance becomes marginal in the setting of hilar strictures. In a recent meta-analysis of 32 studies, including 1123 patients with cholangiocarcinoma (CCA), EUS-fine needle aspiration (FNA) showed significantly better diagnostic sensitivity (73.6%) than ERCP fluoroscopic biopsies (56.0%).^67^ Endoscopic ultrasound-FNA of distal bile duct strictures tends to have higher sensitivity than hilar/proximal, estimated at 50% to 89%.^68,69^ In liver transplant candidates, experts caution against transperitoneal biopsies due to the potential risk of tumor seeding and peritoneal dissemination.^70,71^

A tailored and personalized approach is recommended when tackling IDBS. Since visual inspection using direct cholangioscopy is marred by interobserver subjectivity, tissue acquisition becomes crucial for accurate diagnosis. Subsequent treatment is contingent on the institutional expertise vis-à-vis the stricture characteristics (proximal vs. distal and intrinsic vs. extrinsic) and the patient’s preference in the face of associated comorbidities.

## Conclusion

Benign biliary strictures continue to pose a clinical challenge in at-risk patients. Reviewing good cross-sectional imaging before embarking on endoscopic intervention is paramount to reducing the prospect of missed malignancy. Occasionally, and in indeterminate cases, longitudinal follow-up is necessary to ensure a benign clinical course. endoscopic retrograde cholangiopancreatography-guided balloon dilation with or without multiple plastic stent placements is the first-line therapy. Although FC-SEMS can be considered in select cases, this practice remains off-label in the USA. Surgery is reserved for refractory or failed instances. A multi-disciplinary approach, including radiology, interventional radiology, advanced endoscopy, and biliary surgery, is often necessary but is always welcome to tailor treatments for specific scenarios.

## Figures and Tables

**Table 1.** Reviews of the Bismuth Classification of Benign Biliary Strictures

Bismuth Class	Criteria
I	>2 cm distal to hepatic confluence
II	<2 cm distal to hepatic confluence
III	At the level of the hepatic confluence
IV	Involving the right or left hepatic duct
V	Extending into the left or right hepatic branch ducts

**Table 2.** Reviews of the Strasberg Classification for Benign Biliary Strictures

Strasberg Class	Criteria
A	Cystic duct leaks or leaks from small ducts in the liver bed
B	Occluded right posterior sectoral duct
C	Bile leak from divided right posterior sectoral ducts
D	Bile leak from main bile duct without major tissue loss
E1	>2 cm distal to hepatic confluence
E2	<2 cm distal to hepatic confluence
E3	Hilar stricture with the hepatic confluence preserved
E4	Hilar stricture with involvement of the hepatic confluence
E5	Combined common hepatic duct and aberrant right hepatic duct injury
